# Mirror-mediated string-pulling task in Eurasian jays (*Garrulus glandarius*)

**DOI:** 10.1007/s10071-021-01590-5

**Published:** 2021-12-16

**Authors:** Luigi Baciadonna, Francesca M. Cornero, Nicola S. Clayton, Nathan J. Emery

**Affiliations:** 1 Biological and Experimental Psychology, School of Biological and Chemical Sciences, Queen Mary University of London, Mile End Road, London, E1 4NS UK; 2 Department of Psychology, University of Cambridge, Downing Street, Cambridge, CB2 3EB UK; 3 Department of Life Sciences and Systems Biology, University of Turin, Turin, Italy

**Keywords:** Mirror studies, Avian cognition, Comparative cognition, Corvids, String-pulling

## Abstract

Mirror tasks can be used to investigate whether animals can instrumentally use a mirror to solve problems and can understand the correspondence between reflections and the real objects they represent. Two bird species, a corvid (New Caledonian crow) and a parrot (African grey parrot), have demonstrated the ability to use mirrors instrumentally in mirror-mediated spatial locating tasks. However, they have not been challenged with a mirror-guided reaching task, which involves a more complex understanding of the mirror’s properties. In the present study, a task approximating the mirror-guided reaching task used in primate studies was adapted for, and given to, a corvid species (Eurasian jay) using a horizontal string-pulling paradigm. Four birds learned to pull the correct string to retrieve a food reward when they could see the food directly, whereas none used the reflected information to accomplish the same objective. Based on these results, it cannot be concluded whether these birds understand the correspondence between the location of the reward and its reflected information, or if the relative lack of visual-perceptual motor feedback given by the setup interfered with their performance. This novel task is posited to be conceptually more difficult compared to mirror-mediated spatial locating tasks, and should be used in avian species that have previously been successful at using the mirror instrumentally. This would establish whether these species can still succeed at it, and thus whether the task does indeed pose additional cognitive demands.

## Introduction

Mirrors are a standard tool in comparative cognition: they are used to investigate both mirror self-recognition (MSR) and how non-human animals process mirrored information to accomplish tasks like object manipulation and to obtain visually inaccessible food (see Baciadonna et al. 2021 for a recent review). In seminal studies of MSR, chimpanzees exposed to mirrors for the first time initially reacted to their reflection socially, as if it were a conspecific. Over time, the chimpanzees spontaneously began to display contingent behaviours, and to explore parts of their body not otherwise visible (Gallup 1968; 1970). When subsequently tested on a formal mark test, such subjects were able to locate and remove a mark on their body using the reflection provided by the mirror (Gallup 1970). Regardless of the ecological and methodological validity of the mark test (e.g. Heyes 1994; De Veer and Van Den Bos 1999; de Waal 2019; Vonk 2019), several (mainly large-brained) species such as primates (for a review see Anderson and Gallup 2015), dolphins (Reiss and Marino 2001), elephants (Plotnik et al. 2006), and some corvids (Prior et al. 2008; Clary and Kelly 2016; Buniyaadi et al. 2020) have shown behavioural patterns in line with passing this test.

Less frequently have animals’ abilities to process reflected information been explored separately from MSR. Mirror use investigations are often performed when species fail the mark test, to investigate whether general difficulties in processing mirrored information can explain these negative results. Such instrumental mirror use tasks can be categorised into four types (in addition to mirror image stimulation, usually applied prior to the mark test): mirror-triggered search, mirror-mediated object discrimination, mirror-mediated spatial locating, and mirror-guided reaching (Menzel et al. 1985; Povinelli 1989; Pepperberg et al. 1995). In mirror-triggered search, the mirror is a cue to trigger searching behaviour, for either a food item or a positively rewarded stimulus (Anderson 1986; Povinelli 1989; Pepperberg et al. 1995; Broom et al. 2009; Howell and Bennett 2011; Gieling et al. 2014; Wang et al. 2020b); for instance, a location that is only visible with the use of a mirror may be baited with a food reward, and what is observed is whether a subject looks for the food reward in real space after only seeing its reflection. In this task, therefore, the mirror is simply used as a cue to initiate a search, and therefore, it does not require an understanding of the relationship between the object’s location in space and its reflection. In fact, the subject might not be able to find the food when it is subsequently moved to a new location. In the mirror-mediated object discrimination task, the subject can choose between options (e.g. positive vs. negative stimuli) using the mirror, correctly seeking a positive item and avoiding to seek a negative one, and can do so even when the stimuli are placed in a new location (Menzel et al. 1985; Pepperberg et al. 1995); for example, the location only visible in the mirror may either be baited with a food reward, which the subject should seek, or an aversive or scary object, which the subject should accordingly react to or move away from. In this case, it is assumed that the subject can grasp the correlation between a real object and its reflection, by appropriately reacting to the identity of the stimulus visible only in the mirror. In the mirror-mediated spatial locating, in which the reward is hidden in one of multiple possible locations, and its location is visible only in the mirror, and mirror-guided reaching tasks, in which the subject can only obtain the reward visible in the mirror by also monitoring its own reaching or grasping efforts in the mirror, the subject is additionally required to understand the correspondence between the location of the object in real space and its reflected information (Menzel et al. 1985; Anderson 1986; Povinelli 1989; Pepperberg et al. 1995; Medina et al. 2011). In the case of mirror-guided reaching, the individual should be able to monitor its own movements and the movement of objects, as reflected by the mirror, moment by moment (Menzel et al. 1985; Anderson 1986; Itakura 1987; Povinelli 1989). These different types of mirror tasks require processing mirrored information at different levels, and therefore, form a valuable and standardised way to compare performance across different species (Pepperberg et al. 1995).

A plethora of studies have investigated MSR in large-brained birds such as corvids and parrots (see Derégnaucourt and Bovet 2016; Brecht and Nieder 2020; Baciadonna et al. 2021 for recent reviews), with mixed results: only two out of five magpies (Prior et al. 2008), four out of six Indian crows (Buniyaadi et al. 2020) and six out of ten Clark’s nutcrackers (Clary and Kelly 2016) have been reported to show evidence of MSR. Some potential evidence for MSR were also found in California scrub jays, in which individuals showed decreased levels of cache-protection behaviours when caching in front of a mirror than when caching in front of a live, potentially pilfering conspecific (Dally et al. 2010). Other studies failed to find evidence of MSR in birds (jackdaws, Soler et al. 2014; magpies, Soler et al. 2020; Goffin’s cockatoos and keas, van Buuren et al. 2018; crows, Vanhooland et al. 2019; Brecht et al. 2020; California scrub jays, Clary et al. 2020; azure-winged magpies, Wang et al. 2020b). Only three studies have systematically assessed instrumental mirror use (Pepperberg et al. 1995; Taylor et al. 2010; Medina et al. 2011), but never in direct association with MSR. In one study in which MSR and one mirror use task were jointly investigated, the azure-winged magpies did not pass either task (Wang et al. 2020b). However, some species that fail the mark test are able to use the mirror instrumentally, suggesting that MSR does not seem to be essential for instrumental mirror use and vice versa (Povinelli 1989; Heschl and Burkart 2006).

Of the avian species in which mirror use has been systematically studied, grey parrots were tested more extensively, in a study using mirror-triggered search, mirror-mediated object discrimination, and mirror-mediated spatial locating tasks (Pepperberg et al. 1995): both parrots tested, Alo and Kyaaro, successfully approached positive stimuli and moved away from negative stimuli. Their ability to discriminate objects also generalised to novel stimuli, suggesting the behaviour was mirror-mediated object discrimination rather than mirror-triggered search, and that parrots were reacting to the actual reflection of the objects, rather than learning to behave in a specific way when seeing a specific stimulus (Pepperberg et al. 1995). In the mirror-mediated spatial locating task, both birds were able to find hidden food in one of several possible hiding spots, even when a new setup and additional locations were presented, suggesting that the reflective properties of the mirror had indeed been utilised (Pepperberg et al. 1995). Similarly, when New Caledonian crows were tested in a four-box spatial locating task, all four subjects were able to find the hidden food (in 2–3 blocks of 10 trials; Medina et al. 2011).

However, to date, corvids and parrots have not been tested in a mirror-guided reaching task (Menzel et al. 1985; Itakura 1987; Povinelli 1989), because birds do not have arms or hands to explore their body or to reach for food or objects, making the execution of this task complicated. The patterned string-pulling task has been proposed as a potential way to test birds’ mirror-guided reaching abilities and to make comparisons with primates (Pepperberg et al. 1995). Although the patterned string-pulling task has a long history in comparative cognition (see Obozova et al. 2014; Jacobs and Osvath 2015 for extensive reviews), this task has scarcely been used to investigate complex forms of mirror use in birds. An exception involves a study conducted with New Caledonian crows (Taylor et al. 2010): crows were tested in a vertical string-pulling task to disentangle, primarily, between perceptual motor feedback or insight as the cognitive mechanisms that might be involved in success on this task. However, four birds were tested in a condition in which a mirror was introduced to provide more visual information during a visually restricted string-pulling task. In the visually restricted string-pulling task, the view of the suspended bait was occluded by a wooden platform. The birds had to look through a small hole placed in the centre of the platform to pick the correct string. In the mirror condition, a small mirror was placed on the side of the platform, allowing the birds to manipulate the string while seeing their reflection and the reward or stone attached to the strings. Only two of the four birds in the mirror condition solved the task: one bird oriented its head or body towards the mirror in the successful trials, whereas the other succeeded regardless of its body/head position (Taylor et al. 2010). The initial assumption was that naïve crows could actually benefit from using the mirror to gather information about the position of the reward and to monitor their movements, but the results suggest that trial and error, mediated by perceptual motor feedback, is the main mechanism adopted by these crows to solve the patterned string-pulling task.

Although some evidence suggests that at least some jays (California scrub jays, Dally et al. 2010) may be capable of mirror self-recognition, it is not known whether any jay species can use mirrors instrumentally. Like scrub jays, Eurasian jays cache food for the winter and primarily breed in territorial pairs. Several studies have shown that they possess advanced cognitive abilities, such as object permanence (Zucca et al. 2007, Salwieczek et al. 2009), understanding causal relationships between objects (Davidson et al. 2017), flexible adjustment of cache-protection strategies to avoid pilfering by other birds (Shaw and Clayton 2013, 2014; Legg and Clayton 2014; Legg et al. 2016), tool-use in captivity (Cheke et al. 2011; Amodio et al. 2019), and desire-state attribution towards conspecifics (Ostojić et al. 2013, 2014, 2016). However, to date, Eurasian jays have not been formally tested using either the mark test or instrumental mirror use. Testing their performance on these tasks, therefore, represents an excellent opportunity to investigate how jays process mirror information, before exploring whether Eurasian jays are able to understand the nature of their own reflection, as California scrub jays possibly do. The aim of this study was to explore instrumental mirror use through a task conceptually similar to a mirror-guided reaching task, but adapted for birds. For this reason, a modified version of the string-pulling task was used, in which the jays had to understand the correspondence between the real position of objects in space and their reflection to obtain the reward. We hypothesised that birds who could link information on the position of the reward in space with its reflection would be able to pull the correct, baited string.

## Methods

### Subjects and housing conditions

Between September and November 2019, eight adult Eurasian jays were tested, from two aviary groups: five from Aviary I (Caracas, Lima, and Lisbon, males; Washington and Wellington, females, all 13 years old) and three from Aviary II (Hoy and Romero, males; Hunter, female, all 14 years old). Outside of testing, jays lived in groups in two large outdoor aviaries (20 × 6 × 3 m). Smaller indoor compartments (3 × 1 × 2 m) connected to the aviary by hatch doors (0.5 × 0.5 m) were accessible to the jays. These indoor testing compartments were used as testing spaces by shutting the hatch doors that connected to the aviaries. Subjects participated voluntarily, and they were tested for no longer than 15 min/day. During testing, each individual was physically and visually isolated from other jays. Birds were food restricted an hour before testing (birds were never food restricted more than 4 h/day, and water was provided ad libitum). Outside of testing, birds were fed a maintenance diet of soaked cat biscuits, vegetables, seeds, fruit, and hard-boiled eggs. The jays were hand-raised by licensed breeders and had subsequently lived in laboratory settings. They had been involved in several experiments by the time they participated in this study and were, therefore, fully habituated to testing and video-recording procedures. These jays had also received prior mirror exposure during a series of mirror-stimulation experiments. These tasks did not require use of the mirror, and were designed to motivate the birds to spend time in front of the mirror and observe its effects and potentially their movements when reflected by the mirror. These tasks included the placement of a mirror inside their aviary for 2 weeks; a mirror preference test in which they could choose to approach either a mirror or cardboard surface to retrieve a preferred food (mealworms, waxworms or dry cat food) or remove a layer of cling film in front of the mirror before obtaining food (Baciadonna et al. in prep). All the procedures involving animal handling and treatment were approved by the University of Cambridge (ZOO63/19) and followed Home Office Regulations and the ASAB’s Guidelines (Association for the Study of Animal Behaviour 2020).

### Apparatus

**Fig. 1:**
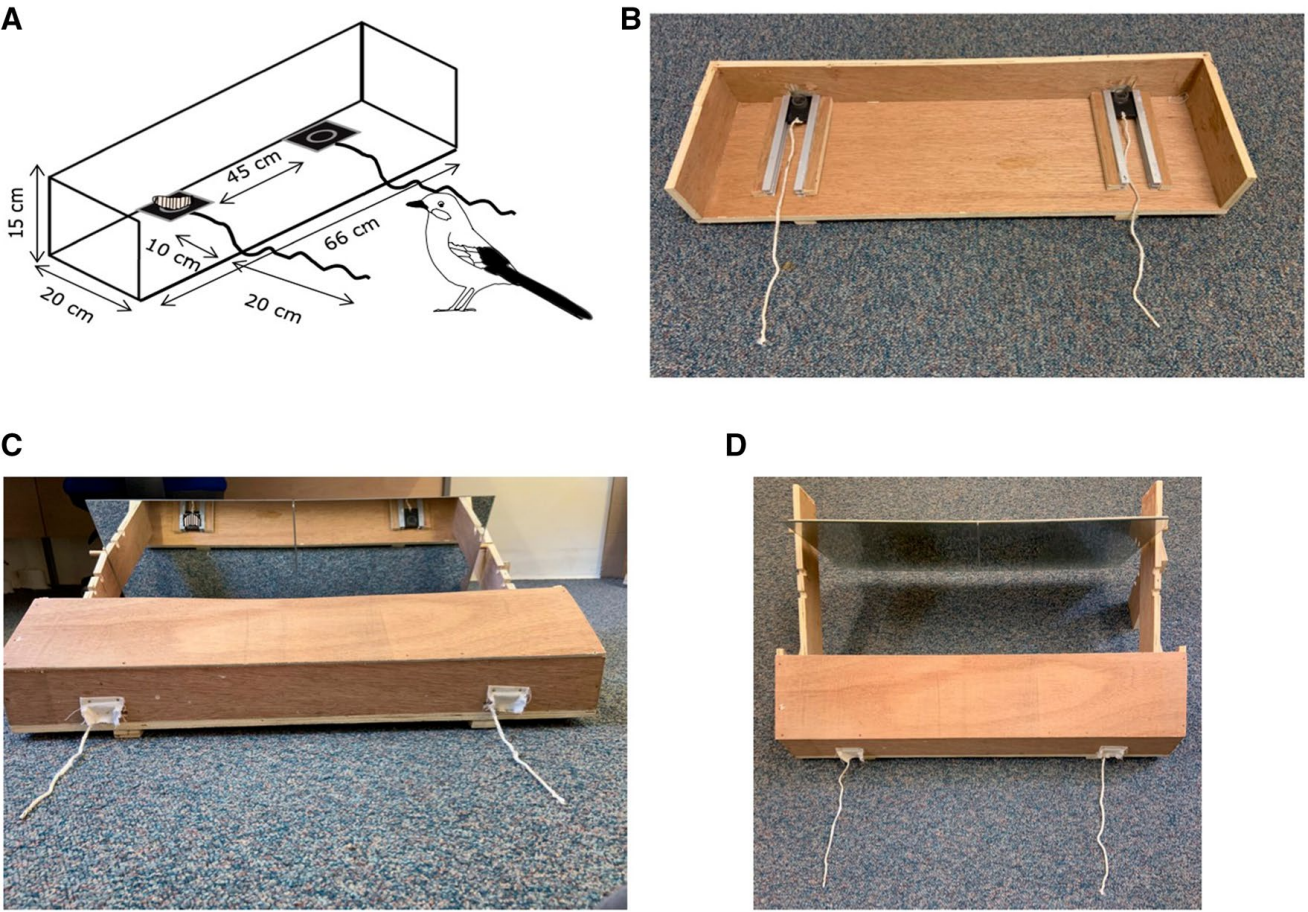
**A** Representation of the apparatus used in the Experiment 1 with the relative measures. **B** Representation of the frontal view of the apparatus used in the Experiment 1. **C** Representation of the frontal view of the apparatus used in the Experiment 2, including cartoon waxworm depicted on the left black plastic plate. The apparatus was a modified version of the one used in the Experiment 1. The apparatus was rotated and two small openings were made for plastic plates containing the reward to pass through. These openings were covered with small pieces of opaque white cloth to hide the location of the rewarded plate from the bird’s view. An angled wooden barrier was fixed on top of the three wooden sides to cover the same view from the top and lateral sides. Two adjacent mirrors (30 × 30 cm) were fixed to the apparatus using a wooden frame, so that they hung at an angle (60°). The birds can see the position of the waxworm placed in the black plate only using the reflections provided by the mirror. **D** Representation of the top view of the apparatus used in the Experiment 2

For Experiment 1, the apparatus (Fig. 1a, b) consisted of a rectangular wooden base (66 × 20 cm) with three wooden sides (two 20 × 8 cm and one 66 × 8 cm) and one open long side. There were two sliding systems mounted on the wooden base, on which two black plastic plates (4.5 × 4.5 cm) were lodged; the distance between the two sliding plates was 45 cm. In the middle of each plate, a small Perspex tube (Ø 2 × 0.5 cm) was fixed to hold a waxworm. The two plates were connected to strings (total length 30 cm, 20 cm of which rested outside the apparatus) allowing the birds to pull the plates towards them to consume the reward. The apparatus was placed against the wire mesh of one compartment, with the open side facing the birds. A small opening was created in this wire mesh, allowing the jays to retrieve the reward once they pulled it closer.

For Experiment 2, a modified version of the apparatus used in Experiment 1 (Fig. 1c, d) was used. The apparatus was rotated and two small openings (4.5 × 1 cm) were made on the long wooden side (66 × 8 cm), leaving space for the plastic plates to fit through. These openings were occluded with small pieces of opaque white cloth to cover the location of the rewarded plate from the bird’s view. An angled wooden barrier was fixed on top of the three wooden sides, to cover the same view from the top and lateral sides. Two adjacent mirrors (30 × 30 cm) were fixed to the apparatus using a wooden frame, so that they hung at an angle (60°). The distance between the edge of the angled wooden barrier and the edge of the mirror was 20 cm. This ensured birds could only see the plastic plates and any rewards in the mirror, and not directly.

### General procedure

#### Training

Before Experiment 1, training was performed to ensure that the subjects knew they would only obtain the reward by pulling the string, using the apparatus displayed in Fig. 1a, b. In this phase, both plastic plates were baited with one waxworm. The strings were arranged parallel to one another and perpendicular to the front of the apparatus. Initially, the plastic plates were set close to the wire mesh, so the birds could reach the reward by slightly moving the plate or pulling the string. Later, the plates were moved back 10 cm from the edge of the mesh (as in Experiment 1 and 2) and the birds could only obtain the reward by pulling the string. Once birds obtained one reward successfully by pulling the string at this point, they were considered ready for Experiment 1.

#### Experiment 1

Experiment 1 was similar to the training phase. However, only one plate was baited with a waxworm, whereas the other remained empty. The baited side was randomised across trials, but the same side was not baited more than twice in a row. Each bird had to make a correct choice on an average of 80% of trials across two consecutive blocks of 10 trials to pass the test. If birds did not approach the apparatus for four consecutive days, they were not tested further.

#### Experiment 2

Experiment 2 was performed using the apparatus displayed in Fig. 1c and d. As in Experiment 1, only one plate was baited with a waxworm. In Experiment 2, however, the plates and the position of the bait were only visible by looking at the mirrors. Therefore, the birds had to understand the correspondence between the real location of the baited plate and its reflected information to pull the correct string. Each bird had to make correct choices on an average of 80% of trials across two consecutive blocks of 10 trials to pass the test. If birds did not approach the apparatus for four consecutive days, or if they completed 200 trials before reaching criterion, they were not tested further.

#### Analyses

Experiments 1 and 2 were filmed using a GoPro^®^ Hero 4 video-camera. Subjects’ choices for each trial were recorded live on a pre-made sheet as 0 (incorrect) or 1 (correct). A Laterality Index was calculated on the correct trials using the formula (L − R)/(L + R). Positive values indicated a preference for the left side and negative values indicated a preference for the right side. *Z* scores were calculated using the formula *z* = (*R* − 0.5 *N*)/√(0.25 *N*), where *R* equals the number of correct responses and *N* indicates the sum of right plus left choices, to establish statistical significance. Subjects with a positive *z* score value ≥ 1.96 were considered to have a right-side bias, while those with a negative *z* score value ≤ − 1.96 were considered to have a left-side bias. Subjects scoring between the two values were considered unbiased.

### Results

#### Training

Seven out of eight birds were able to retrieve the reward by pulling the strings at least once (range of sessions, 3–14), and therefore, were eligible for Experiment 1. Hunter stopped approaching the apparatus after 17 days and was excluded from further testing.

#### Experiment 1

**Fig. 2:**
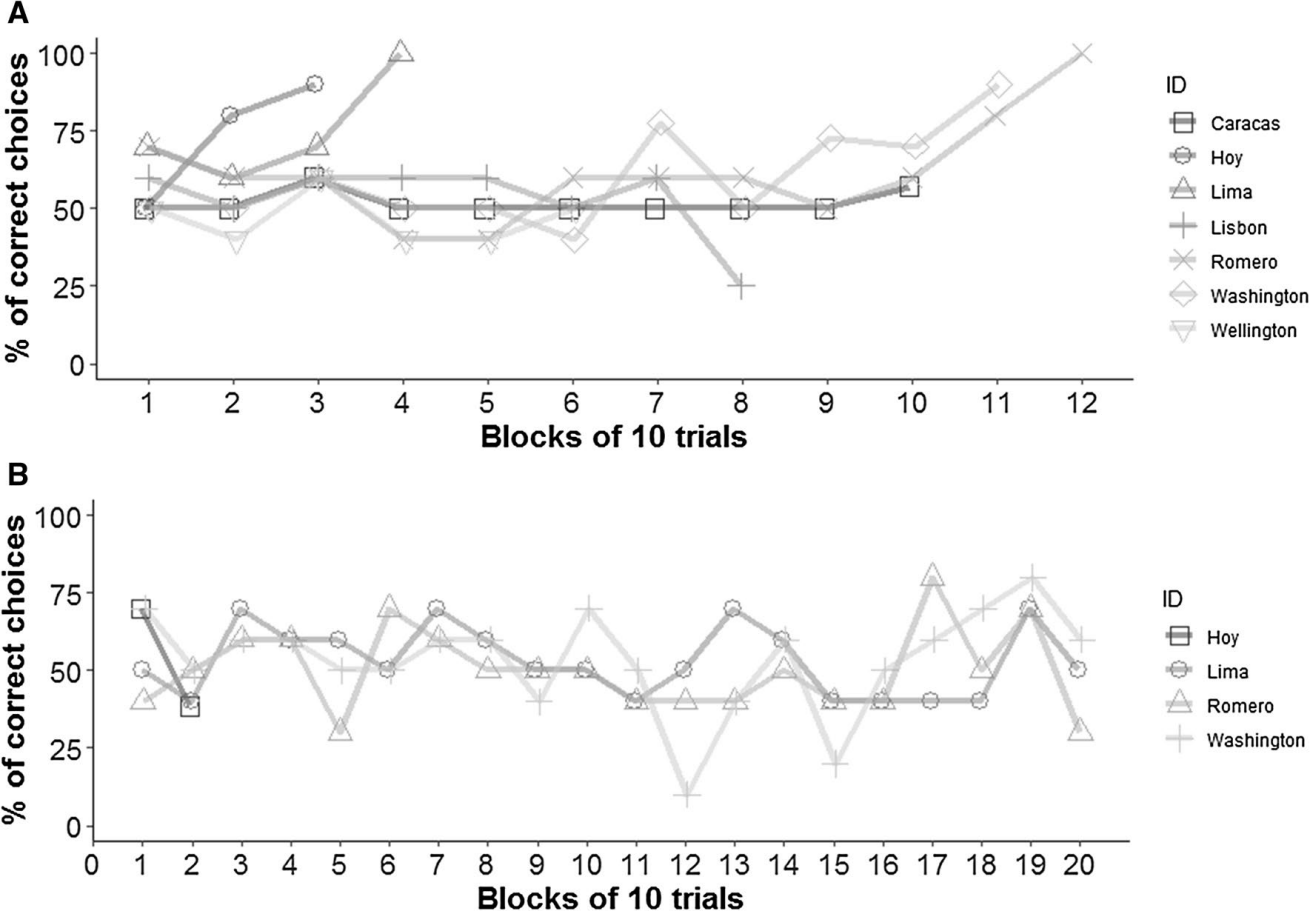
**A** Percentage of correct choices across trials (block of 10 trials) for each jay in Experiment 1. Only Lima, Washington, Hoy, and Romero passed the task by reaching the pre-established criterion **B** Percentage of correct choices across trials (block of 10 trials) for each jay in Experiment 2. None of the birds reached the pre-established criterion

Four jays (Lima and Washington from Aviary I; Hoy and Romero from Aviary II) passed the task by reaching the pre-established criterion (Fig. 2a) whereas the other three birds (Caracas, Lisbon, and Wellington) stopped approaching the apparatus for four consecutive sessions and were excluded from further testing. The birds’ performance is reported in Fig. 2a. Caracas and Wellington preferentially pulled the right string, whereas Romero had a left side bias overall, but he eventually passed the test. Hoy, Lima, Lisbon, and Washington had no side bias (Table 1).

**Table 1:** Overview of the jays’ performance in Experiment 1: total number of trials completed and whether the jays passed the criteria (average of 80% correct over two consecutive sessions), number of correct strings pulled and associated Laterality Index, *Z* score and presence of side bias

ID	Aviary	Sex	Total number of trials	Success	Number of correct string pulls		Laterality Index	*Z* score	Side bias
					Right	Left			
Caracas	Aviary I	M	97	Failed	46	4	− 0.84	5.93	Right
Lima	Aviary I	M	40	Passed	14	16	0.06	− 0.36	No bias
Lisbon	Aviary I	M	74	Failed	23	18	− 0.14	0.78	No bias
Washington	Aviary I	F	110	Passed	38	28	− 0.15	1.23	No bias
Wellington	Aviary I	F	52	Failed	22	2	− 0.83	4.08	Right
Hoy	Aviary II	M	30	Passed	10	12	0.09	− 0.42	No bias
Romero	Aviary II	M	120	Passed	22	54	0.4	− 3.48	Left

#### Experiment 2

The jays that passed Experiment 1 were tested on Experiment 2: none passed. Hoy stopped approaching the apparatus for four consecutive sessions; Lima, Washington, and Romero reached 200 trials without reaching criterion. The birds’ performance is reported in Fig. 2b. Lima and Washington preferentially pulled the string on the right side, whereas Hoy and Romero had no bias (Table 2).

**Table 2:** Overview of the jays’ performance in Experiment 2: total trials completed and whether the jays passed the criteria (average of 80% correct over two consecutive sessions), number of correct strings pulled and associated Laterality Index, *Z* score and presence of side bias

ID	Aviary	Sex	Total number of trials	Success	No of correct string pulls		Laterality Index	*Z* score	Side bias
					Right	Left			
Lima	Aviary I	M	200	Failed	82	24	− 0.54	5.63	Right
Washington	Aviary I	F	200	Failed	78	29	− 0.45	4.73	Right
Hoy	Aviary II	M	23	Failed	7	5	− 0.12	0.57	No bias
Romero	Aviary II	M	200	Failed	45	55	0.10	− 1	No bias

## Discussion

The aim of this study was to test instrumental mirror use in Eurasian jays by presenting a modified version of the string-pulling task. Jays had to understand the direct link between the reward’s position in space and the reflection provided by the mirror to pull the correct string. This is the first investigation approaching a mirror-guided reaching task in birds. After an initial familiarisation with the string-pulling task, four out of seven birds learned to obtain a reward by pulling the correct string. However, when the baited and un-baited strings could only be seen when reflected by a mirror, and not directly, none of the jays tested pulled the baited string at significant levels.

The results of Experiment 1 are comparable to those available from several species of corvids tested on a horizontal patterned string-pulling task (Obozova et al. 2014; Jacobs and Osvath 2015). Although the criteria for passing and the number of trials conducted across studies are not identical, what emerges is that the number of subjects that pull the string in a goal-directed manner (in a perpendicular string pattern) is quite small. In a seminal study, all three ravens presented with horizontal strings first touched baited and un-baited strings at chance, though they preferentially then switched to obtain baited strings (Heinrich 1995). Three out of four hooded crows and ravens (Bagotskaya et al. 2012) solved the parallel string pattern within 32 trials, whereas only one California scrub-jay out of five (Hofmann et al. 2016), and one oriental magpie out of eight (Wang et al. 2020a) were able to successfully pass this test (within 50 and 30 trials, respectively). A modified version of the parallel configuration was necessary to increase the number of California scrub jays able to pull the correct string (Hofmann et al. 2016): the location of the reward was made more visible and with higher contrast compared with the unrewarded string; four out of five birds managed to pass this re-designed task within 50 trials. Parrots were more successful in string-pulling tasks in various patterns, however, they were mainly tested using a vertical configuration (Werdenich and Huber 2006; Schuck-Paim et al. 2009; Krasheninnikova and Wanker 2010; Krasheninnikova 2013; Krasheninnikova et al. 2013; Krasheninnikova and Schneider 2014; Molina et al. 2019; Ortiz et al. 2019, see van Horik and Emery 2016 for use of an horizontal variation). Although the vertical version appears to be more challenging than the horizontal configuration, there are some exceptions. In one study, none of the grey parrots passed the horizontal parallel and more complex patterned configurations, though they passed a vertical string-pulling task (Jacobs and Osvath 2015; Molina et al. 2019). In our study, Hoy and Lima were able to pass the task within 30 and 40 trials, whereas Washington and Romero passed in 110 and 120 trials, respectively. Romero also had an overall left side bias, especially during the initial trials. Due to the stringent criteria we applied, the range of trials required for each bird to pass was quite wide, although two birds did pass the test within a similar number of trials to those in other studies. The three other birds stopped pulling the string over four consecutive sessions, performing a range of 52 to 97 trials, with two of them preferentially pulling the string on the right side (Caracas and Wellington).

Limited conclusions can be drawn about causal reasoning in the jays that passed, as it was beyond the purpose of this study to disentangle the different cognitive mechanisms involved in the string-pulling task, which would have required additional configurations (Jacobs and Osvath 2015). Establishing whether jays used goal-directed behaviour in this study is also very difficult in the absence of control experiments testing whether the birds can solve the string-pulling task without relying on proximity with the reward, whether they can generalise the rule to modified versions of the task, and whether their performance on the task is independent of perceptual cues (Schuck-Paim et al. 2009; Jacobs and Osvath 2015). However, the performance of at least four of the jays tested here, those that passed, may be consistent with some understanding of causal reasoning and connectivity being present in Eurasian jays, which may warrant direct investigation in the future studies.

Experiment 2 was performed with only the four jays that successfully completed the horizontal parallel string-pulling task, to investigate whether they could solve a complex visual spatial task using mirrored information. The task presented here, inspired by mirror-guided reaching tasks, is technically and qualitatively different from the tasks developed for primates, to account for the morphological differences between species, but is conceptually similar (Pepperberg et al. 1995). When tested in a mirror-guided reaching task, chimpanzees were able to track the movements of their hands towards the food or object by looking at the mirror, as well as a live video in some conditions, even when the object was reversed laterally, inverted by 180°, or both reversed and inverted (Menzel et al. 1985). Control experiments indicated that two chimpanzees distinguished between pre-recorded and live videos. This level of proficiency was also evident in a subsequent study, in which four macaques were tested in a mirror-guided reaching task (Anderson 1986). Only two macaques out of four were able to use the mirror to locate food and guide their hands to it. Well-designed controls were performed to make sure that the animals realised the association between their hands’ movements and the reflection, and subjects that showed understanding of this relationship behaved accordingly. In trials in which no food was hidden, when the usual movements were performed by the experimenter and the mirror was available, subjects reduced their searching behaviour, whereas they tried to search for the food more often when the mirror was removed. In contrast, the two subjects that were not able to use the mirror instrumentally searched for the food equally in both mirror and mirror-less, un-baited conditions.

A few bird species tested in a similar, mirror-mediated spatial locating task had to use a mirror to locate the bait in one of three or four adjacent locations, normally placed below a countertop. Parrots performed quite well (using three compartments, above 75% correct), even on transfer tests (four compartments, above 60%; and with an overhead mirror, 75%; Pepperberg et al. 1995). New Caledonian crows were tested on a similar task after receiving initial training on a two-box apparatus (three birds performed below 50% correct, but one bird made 90% correct choices over 30 trials): in a four-box apparatus, all the birds (10/10 correct for one crow, with 6/10 trials in the three-choice task) were able to pick the bait correctly within 20–30 trials. The crows’ results are interesting because they were better able to locate the bait in a four-box than a two-box apparatus (Medina et al. 2011). One plausible explanation for this is that they learned over time to associate the location of the food and the image reflected in the mirror. According to the definition of mirror-mediated spatial locating, the subjects must form a mental representation that the stimulus (food or object) reflected in the mirror is the same stimulus located in real space (Povinelli 1989; Medina et al. 2011). Accordingly, it is very likely that the crows (at least three out of four subjects) did not comprehend the use of the mirror in such a fashion. However, they did not simply use the mirror as a trigger to initiate searching behaviour, either (i.e. they selected the correct compartment in the four-box apparatus).

The task conducted in our study cannot be classified as a mirror-guided reaching task because the birds did not need to constantly monitor the movement of the strings to obtain the reward successfully. However, it is still more complex than the mirror-mediated spatial locating task performed with grey parrots and New Caledonian crows (Pepperberg et al. 1995; Medina et al. 2011). In fact, jays could not associate the food with their own image (which they could not see) moving towards the mirror and the correct location, because they needed to use a string to pull the food towards them instead (although, had they been successful, they may have learned to associate the side in which the food was visible with the correct string instead—had this been the case, further configurations, such as crossed-string paradigms, could have been further used to disentangle the mechanisms behind their success). Furthermore, to get the reward, they had to understand that this was possible only using the string, and that the image reflected in the mirror represented both the same string whose end they could see in front of them, as well as the real reward. Moreover, the visual-perceptual motor feedback intrinsically present in the classical string-pulling task is limited in our study. The food is completely hidden apart from in the mirror, and the movement of the string attached to the food cannot be monitored because as it is being pulled it moves out of view of the mirror (Taylor et al. 2010). In our study, birds would need to look to a different, possibly non-intuitive initial location of the food in Experiment 2 (slightly above them, as reflected by the suspended mirror), assume connectivity of a string to the food, a connectivity that cannot be seen directly in front of them, and disregard the fact the food disappears while the correct string is pulled, to learn to solve Experiment 2 correctly. These disruptions of the visual presentation and subsequent visual feedback afforded by Experiment 1 may have not given the jays enough to go on to learn to use the mirror to solve Experiment 2 during the duration of the study. The performance of the jays, overall, does not indicate that they were able to solve this complex mirror-mediated spatial locating task. Based on their performance, it is, however, possible to suggest that they used the mirror as a trigger to initiate searching behaviour. For example, a mirror-mediated spatial locating task recently performed in azure-winged magpies showed that four out of five birds did not search for the food in the location where it was hidden (i.e. in a compartment above their heads; instead the birds often went behind the mirror), although they looked at the mirror more often when the food was present than when it was not and likely used the mirror as a cue to trigger a search (Wang et al. 2020b). Therefore, the performance of the Eurasian jays could have been due to the limited direct visual-perceptual motor feedback provided, supporting the argument that this feedback is crucial for the solution of string-pulling tasks (Taylor et al. 2010, 2012; Jacobs and Osvath 2015), rather than due to an inability to use mirrored information, although other explanations, discussed as follows, are also possible.

It is possible that other factors implicit in the setup of this novel task, aside from the requirement to use the mirror to find the food, may have accounted for the jays’ behaviour. For example, the limited visual information available in general, aside from the lack of visual-perceptual motor feedback, may account for the birds’ performance. Jays could only see the end of the string protruding from the apparatus, and could see the rest of the string and the reward only reflected in the mirror. Thus, jays may have been unable to understand that connectivity between the string available to them and the string reflected in the mirror was preserved, rather than interrupted. In studies in which “broken strings” were used, birds with previous string-pulling experience tended to select the unbroken strings, whereas naïve birds tended to choose at chance (see a discussion of this in Bastos et al. 2021). It is possible that these experienced Eurasian jays may have expected connectivity as a requirement of the task, and that this lack of visible connectivity may have hindered their performance. After all, many corvid species tested, including Eurasian jays, have shown evidence of fairly sophisticated physical cognition abilities (Emery and Clayton 2009; Cheke et al. 2011), and Eurasian jays have even shown evidence of expecting sufficient physical support for objects in a violation of expectations paradigm (Davidson et al. 2017). Consequently, the jays may have an expectation about the necessity for strings to be connected. In addition, it is possible that other executive functions, such as an inability to inhibit selecting a string directly, without pausing to observe and inspect the mirror, may also account for this failure: the performance of corvid species on delayed gratification tasks is variable (Miller et al. 2019), and inhibiting the choice of a string to obtain more information from the apparatus may have been difficult for the jays.

To our knowledge, this is the first study to investigate a complex form of instrumental mirror use in Eurasian jays, a task that, like previously designed mirror-mediated spatial locating tasks, can be tailored such that solving it would require understanding of mirrored information as depicting a real object in real space. However, detangling the specific mechanisms subjects may use to solve it would require additional controls and configurations than those used here, in which the birds never solved the task. Although this task cannot be categorised as mirror-guided reaching, since the birds did not need to constantly monitor, and re-adjust, the movement of the strings using the mirrored information, it likely adds an additional level of cognitive demand to the classic mirror-mediated spatial locating task, through the requirement to use mediating strings. Although the Eurasian jays’ performance in this task did not reveal an ability to use mirrored information, this study does provide a novel methodology to study how birds may process mirrored information, in a manner more similar to the primate mirror-guided reaching task. Given the additional conceptual difficulties the task may require compared to simpler mirror-guided spatial locating tasks, it would be worth investigating whether other avian species, especially those successful at other mirror use tasks, succeed on this novel task, or whether it poses sufficient additional cognitive demands to hinder their performance as well.
